# Supplementary material for: Genome sequencing and analysis of Salmonella enterica subsp. enterica serovar Stanley UPM 517: Insights on its virulence-associated elements and their potentials as vaccine candidates
Source: PeerJ. 2019 Jun 28;7:e6948. doi: 10.7717/peerj.6948 (PMC6601603; doi:10.7717/peerj.6948)
Supplement: Supplemental Information 1 [file peerj-07-6948-s001.docx]

| **Gene** | **Antibiotic class** | **Resistance mechanism** |
| --- | --- | --- |
| emrR | Fluoroquinolone | Efflux |
| emrB |  | Efflux |
| patA |  | Efflux |
| MdtK |  | Efflux |
| bacA | Peptide | Target alteration |
| PmrF |  | Target alteration |
| CRP | Penam, fluoroquinolone and macrolide | Efflux |
| UhpT | Fosfomycin | Target alteration |
| GlpT |  | Target alteration |
| cpxA | Aminocoumarin and aminoglycoside | Efflux |
| baeR |  | Efflux |
| soxS | Monobactam, carbapenem, rifamycin, penam, triclosan, glycylcyclin, tetracycline, cephalosporin, phenicol, penem, fluoroquinolone antibiotic and cephamycin | Target alteration, reduced permeability to antibiotic, efflux |
| marA |  | Reduced permeability to antibiotic, efflux |
| mdsC | Cephalosporin, penam, monobactam, phenicol, penem, carbapenem and cephamycin | Efflux |
| mdsA |  | Efflux |
| golS | Penam, cephalosporin, monobactam, phenicol , penem, carbapenem and cephamycin | Efflux |
| acrB | Rifamycin, cephalosporin, triclosan, glycylcycline, tetracycline, penam, phenicol, fluoroquinolone | Efflux |
| acrA |  | Efflux |
| soxR |  | Target alteration, efflux |
| marR |  | antibiotic target alteration; antibiotic efflux |
| kdpE | Aminoglycoside | Efflux |
| AAC(6')-Iy |  | antibiotic inactivation |
| mdfA | Rhodamine, tetracycline and benzalkonium chloride | Efflux |
| mbsA | Nitroimidazole | Efflux |
| msrB | Macrolide and streptogramin | Efflux |
| H-NS | Penam, tetracycline, cephalosporin, fluoroquinolone, macrolide and cephamycin | Efflux |
| sdiA |  | Efflux |
